# Spectrophotometric Determination of Cr(III) and Pb(II) Using Their Complexes with 5,11,17,23-Tetra[(2-ethyl acetoethoxyphenyl)(azo)phenyl]calix[4]arene

**DOI:** 10.1155/2015/860649

**Published:** 2015-04-23

**Authors:** Le Van Tan, Tran Quang Hieu, Nguyen Van Cuong

**Affiliations:** ^1^Department of Chemical Engineering, Industrial University of Ho Chi Minh City, 12 Nguyen Van Bao Road, Ho Chi Minh City 70000, Vietnam; ^2^Saigon Technology University, 180 Cao Lo, Ho Chi Minh City 70000, Vietnam

## Abstract

New complexes of 5,11,17,23-tetra[(2-ethyl acetoethoxyphenyl)(azo)phenyl]calix[4]arene (TEAC) with Pb(II) and Cr(III) were prepared in basic solution with a mixture of MeOH and H_2_O as solvent. The ratio of TEAC and metal ion in complexes was found to be 1 : 1 under investigated condition. The complex formation constants (based on Benesi-Hildebrand method) for TEAC-Pb(II) and TEAC-Cr(III) were 4.03 × 10^4^ and 1.2 × 10^4^, respectively. Additionally, the molar extinction coefficients were 5 × 10^4^ and 1.42 × 10^4^ for TEAC-Pb(II) and TEAC-Cr(III), respectively. The H-Point Standard Addition Method (HPSAM) has been applied for simultaneous determination of complexes formation of Cr(III)/Pb(II) and TEAC with concentration from 2 : 1 to 1 : 20 (w/w). The proposed method was successfully utilized to invest lead and chromium contents in plating wastewater samples. The results for several analyzed samples were found to be in satisfied agreement with those acquired by using the inductively coupled plasma mass spectrometry (ICP-MS) technique.

## 1. Introduction

Calixarenes are a product formed from the condensation reaction of* p*-substituted phenols with formaldehyde [1]. Based on these frameworks, scientists have developed a wide range of derivatives by the alkylation of phenolic groups at the lower rim [2–4] or modification of upper rim to form many derivatives. Calixarenes are ideal frameworks for the development of chromogenic ionophores in the molecular recognition of ionic species of chemical and biological interests since the incorporation of a suitable sensory group into the calixarene results in a tailored chromogenic receptor [5]. A variety of compounds based upon calixarene having nitrophenylazophenol, nitrophenol, indoaniline, indophenol, and azophenol functional groups have successfully designed and exhibited a pronounced chromogenic behavior towards Na^+^, K^+^, Cs^+^, Ca^2+^, UO_2_^2+^, and even chiral amines [5–7]. Azocalixarenes are generated by the electrophilic substitution reaction of nitrogen atoms at the* p*-position unit of the calixarene structure, having several isomers based on the position of the nitrogen atoms and the ring size [8, 9]. These compounds consist of at least a conjugated chromophore azo (–N=N–) group and two or more aromatic ring groups, which are important classes of organic colorants under different names such as diazotizated calixarene derivatives, “azocalixarenes” [10]. These compounds have recently attracted great interest in a number of fields such as metal extraction, food technology, and environmental engineering [11, 12]. Therefore, many reports for metal ions were intensively investigated in recent years. The calixarenes had shown applications of optical spectroscopic methods in calixarene chemistry including vibrational spectroscopy, UV-Vis spectrometry, luminescence spectroscopy, ellipsometry, and various optical microscopic methods [13, 14]. Moreover, complexations of azocalixarene with metal ions such as Ni^2+^, Cr^3+^, Fe^3+^, Co^2+^, Pb^2+^, Cu^2+^, Hg^2+^, and Th^4+^ have been studied [1, 11, 15–17].

Lead and chromium are toxic elements for humans and animals. They caused nervous system depressants, cancer risk, and a number of symptoms related to the gastrointestinal tract [18]. Determinations of chromium and lead ions were carried out by many different methods, such as atomic absorption spectroscopy (AAS) and inductively coupled plasma mass spectrometry (ICP-MS). Additionally, the high cost is also a problem with many laboratories. Previously, we have reported simple, fast, sensitive, and spectrophotometric determinations of several metal ions without preconcentration using 5,11,17,23-tetra[(2-ethyl acetoethoxyphenyl)(azo) phenyl]calix[4] arene (TEAC) [19]. In this paper, the application of the simultaneous method (H-Point Standard Addition Method, HPSAM) for analysis of Pb(II) and Cr(III) ions using the azocalixarene (TEAC) has been reported. The effect of parameters on the TEAC-Cr and TEAC-Pb complexes formation was also reported. Additionally, composition of TEAC-Cr and TEAC-Pb was confirmed by ESI-MS.

## 2. Experimental Section

### 2.1. Reagents and Instruments

All chemicals and solvents were of analytical grade and used without further purification unless otherwise mentioned. Double distilled water was degasified before experiment. The preparation and characterization of 5,11,17,23-tetra[(2-ethyl acetoethoxyphenyl)(azo)phenyl]calix[4]arene (TEAC) were reported elsewhere [17].

A Perkin Elmer's Lambda 25 UV/Vis scanning spectrophotometer was used to record the absorbance spectra with 1.0 cm path length quartz cell. The mass spectrum was measured by TSQ LC/MS/MS Triple quadrupole mass spectrometers (Thermo Fisher Scientific, Inc.).

### 2.2. Procedure

Appropriate volumes (0.1 mL to 0.8 mL) of Pb(II) or Cr(III) standard solutions (1 × 10^−3^ M) were added to 2 mL of TEAC (1 × 10^−3^ M) solution in MeOH. 5 mL Na_2_HPO_4_/NaOH buffer solution was then added to the resulting solution to keep the pH in the range of 9-10 and adjusted with a mixed solution of MeOH-H_2_O. Afterward, a portion of the solution was transferred into a 1 cm quartz cell and variations of absorbance were recorded for each sample with TEAC-Cr(III) at 488 nm and TEAC-Pb(II) at 458 nm, respectively.

### 2.3. Binary-HPSAM

For the determination of Pb(II) ions using HPSAM, the synthetic solutions containing Pb(II) ions were prepared with concentration from 0.2 to 5 × 10^−3^ M. Then 5 mL TEAC solution (1 × 10^−3^ M), 5 mL synthetic solution, 5 mL buffer solution (pH = 10-11), and 0.1 mL Cr(III) 10^−3^ M standard solution were mixed in 25 mL volumetric flasks. Absorbance of solution was measured at 420 nm and 480 nm against a reagent blank and graphs of absorbance versus concentration were plotted.

## 3. Results and Discussion

### 3.1. Absorbance Spectra

**Figure 1:**
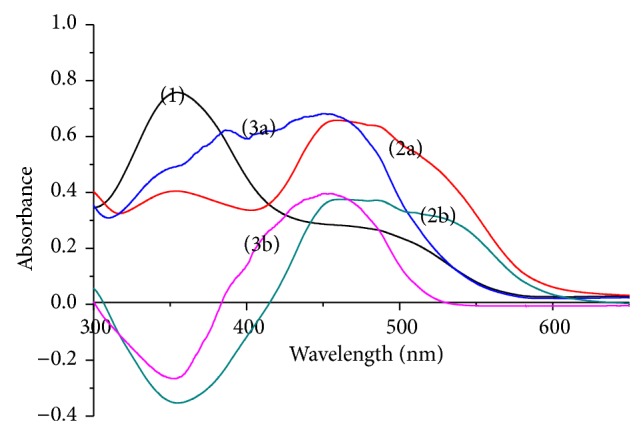
Absorption spectra of TEAC (1), TEAC-Pb(II) (2a), and TEAC-Cr(III) (3a) against methanol-water solvent, TEAC-Pb(II) (2b), and TEAC-Cr(III) (3b) complex against reagent blank.

The absorbance spectra of TEAC-Pb(II) and TEAC-Cr(III) against the blank TEAC were presented in Figure 1. As seen in Figure 1, the TEAC exhibited a maximum absorbance at 355 nm, while addition of lead and chromium ions to TEAC solution led to reducing the absorbance peak at 355 nm and appearing new peaks at 458 nm and 488 nm for lead and chromium complexes, respectively. Additionally, Figure 1 showed that the complexes spectra overlapped with each other. The spectrum of TEAC-Pb(II) appeared symmetrically in the range of 400 nm to 480 nm, while spectrum of TEAC-Cr(III) was stretched. Moreover, the absorbance intensities of the TEAC-Pb(II) complex at 420 nm and 480 nm were similar, while the absorbance intensity of the TEAC-Cr(III) complex was significantly different.

### 3.2. Formation of TEAC-Cr(III) and TEAC-Pb(II) Complexes

#### 3.2.1. Effects of pH

**Figure 2:**
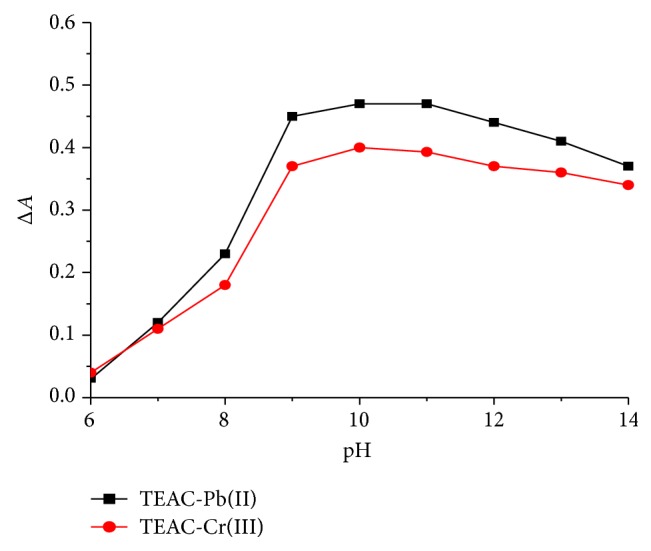
Effect of pH on the change in the absorbance of TEAC-Cr(III) and TEAC-Pb(II) complexes.

The effects of pH on the absorbance of TEAC-Cr(III) at 488 nm and TEAC-Pb(II) at 458 nm are presented in Figure 2 with pH range of 6 to 14. The results indicated that, at the pH values 10-11, the absorbance of the TEAC-Cr(III) complex reached the maximum value, whereas, at the pH values 9–11, the TEAC-Pb(II) complex exhibited the largest absorbance. Therefore, these pH ranges were chosen to conduct further experiments. The effect of buffer solutions (Na_2_HPO_4_/NaOH, Na_2_CO_3_/NaOH, Na_2_B_4_O_7_/NaOH, and NH_3_/NH_4_Cl) on the absorbance of the complexes was examined. The results showed that when using Na_2_HPO_4_/NaOH, the absorbencies of these complexes are stable and achieve the highest value. Therefore, Na_2_HPO_4_/NaOH solution was chosen for future experiments in this study.

#### 3.2.2. Effect of Metal Ion Concentration on Absorption Spectrum and Composition of Complexes

**Figure 3:**
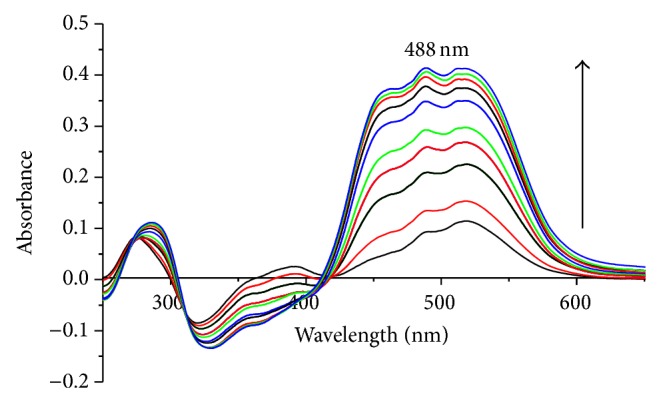
Absorption spectra of TEAC-Cr(III) complex at 488 nm with the increasing concentration of Cr(III) ion from 0.2 × 10^−5^ M to 20 × 10^−5^ M as TEAC blank.

**Figure 4:**
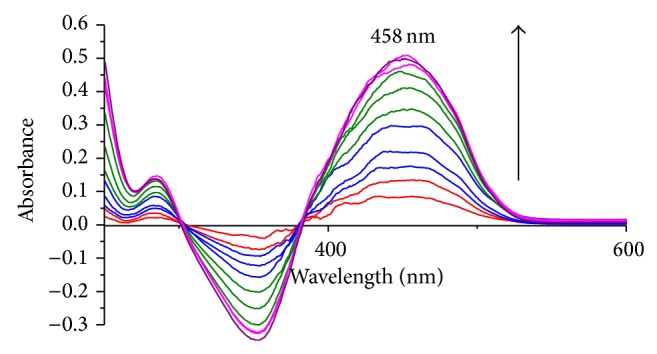
Absorption spectra of TEAC-Pb(II) complex at 458 nm with the increasing concentration of Pb(II) ion from 0.2 × 10^−5^ M to 20 × 10^−5^ M as TEAC blank.

**Figure 5:**
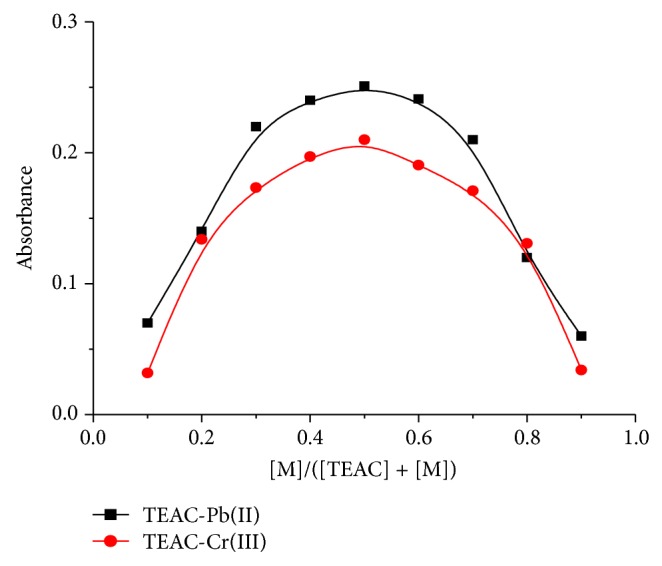
The change in absorbance on ratio of [M]/([TEAC] + [M]) for TEAC-Pb(II) and TEAC-Cr(III) complexes.

The effect of chromium(III) concentration on the absorbance of the TEAC-Cr(III) has been examined at wavelength of 488 nm at pH 10-11 with the TEAC solution as blank. As can be seen from Figures 3 and 4, the absorption spectra of TEAC-Cr(III) complex at 488 nm showed a continuous increase in intensity along with the augment of the chromium(III) concentration in the range of 0.2 × 10^−5^–20 × 10^−5^ M, in which a plateau is reached until the chromium(III) ion concentration increased to 51 × 10^−5^ mol L^−1^. The linear relations between the absorbance and the concentration of chromium(III) were conspicuous in the range of 5 × 10^−6^ to 4 × 10^−5^ M of chromium(III), and linear regression equation was determined to be as follows: absorbance *A* = 0.001 × *C* + 0.2379, *r* = 0.998, and *n* = 6. From the results obtained at Figure 5, it was found that when the concentration ratio [M]/([TEAC] + [M]) was 0.5, the absorbance of the system reached its maximum value. Thus, TEAC formed the complex with Cr(III) and Pb(II) ions with the ratio of 1 : 1 (Figure 5).

**Figure 6:**
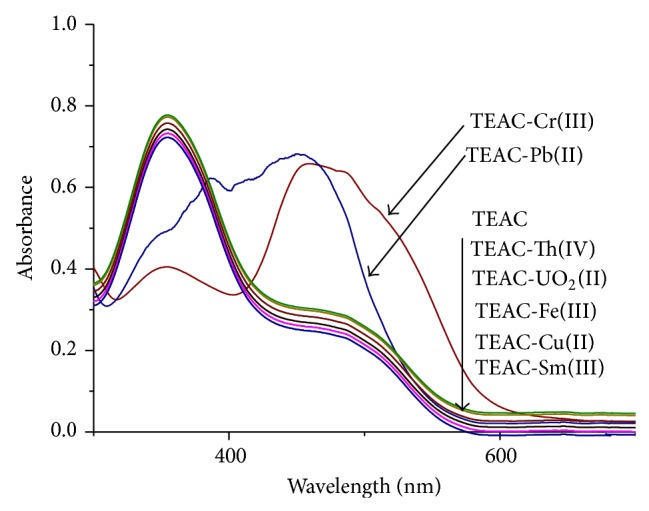
Effect of metal ions on the change in the absorbance of TEAC-Cr(III) and TEAC-Pb(II) complexes with MeOH + H_2_O as solvent and pH = 10.5.

The influence of some other metal ions in complexes formation of TEAC with Cr(III) and Pb(II) was also similarly observed. The absorption spectrum in Figure 6 showed the effect of metal ions such as Th(IV), UO_2_(II), Eu(III), La(III), Sm(III), Ni(II), and Fe(III) on the formation of Cr(III) and Pb(II) complexes. Upon interaction with Cr(III) solution and Pb(II) solution, the reagent TEAC experienced a marked absorption peak at 488 nm and 458 nm, respectively, whereas the addition of other metal ions to the solution of reagent TEAC did not cause any conspicuous change, although their absorption intensities at 365 nm increased or decreased a little compared to free reagent. The reason may be due to the high pH environment; the above metal ions were hydrolyzed or formed solvated complexes with solvents. This phenomenon is very important and showed that reagent TEAC possesses good selectivity towards the presence of chromium(III) and Pb(II) even Th(IV), UO_2_(II), Eu(III), La(III), Sm(III), Ni(II), and Fe(III). The stability constant was estimated by monitoring the decrease in the intensity of the absorbance at the peak with the data reduction being effected using Benesi-Hildebrand plots and the stability constant (*K*) was calculated to be 4.03 × 10^4^ and 1.20 × 10^4^ of TEAC-Cr(III) complex and TEAC-Pb(II) complexes, respectively.

#### 3.2.3. Effect of TEAC Concentration

**Figure 7:**
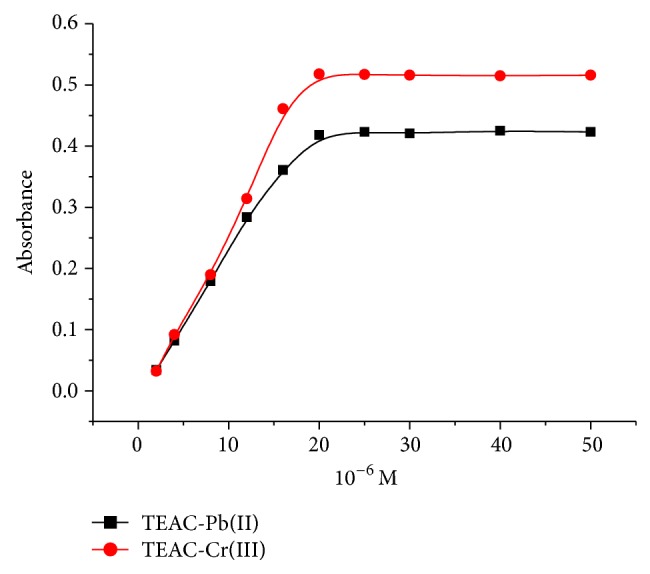
Effect of TEAC reagent concentration on the change in the absorbance of TEAC-Pb(II) and TEAC-Cr(III) complexes.

The effects of TEAC concentration on the absorbance of metal-TEAC complexes were also conducted with concentration of metal ions of 20 × 10^−6^ M, pH = 10-11, and TEAC concentration of 2 × 10^−6^ M to 50 × 10^−6^ M. The absorbencies of TEAC-Pb(II) and TEAC-Cr(III) complexes were measured at 458 nm and 488 nm, respectively. As shown in Figure 7, the increasing TEAC concentration caused an increase in the absorbance since the rise in TEAC concentration led to an increase in Pb(II) and Cr(III) complexes concentration. However, when the TEAC concentration increased to higher than 20 × 10^−6^ M, the concentration of complexes did not change significantly; therefore, the absorbance of complexes did not change.

#### 3.2.4. Stability Constant

The UV absorption spectra of the TEAC-Cr(III) were measured periodically; the results showed that the absorption peak at 488 nm of complex appeared only 5 s after the addition of chromium(III) to the stock solution of the reagent TEAC; the equilibrium was attained within 2 min. The absorbance of the TEAC-Cr(III) and absorbance of the TEAC-Pb(II) are stable up to 90 min and 80 min after complexes formation, respectively. It is proposed that the TEAC could be significant chromogenic ionophores for the recognition of ion chromium(III) and lead(II) and other metal ions [17].

#### 3.2.5. Mass Spectra of Complexes

**Figure 8:**
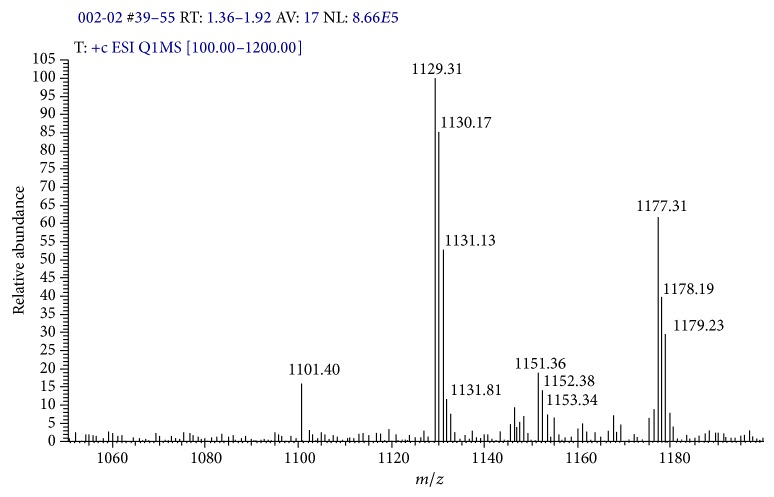
ESI-MS spectra of complex TEAC-Cr(III).

**Figure 9:**
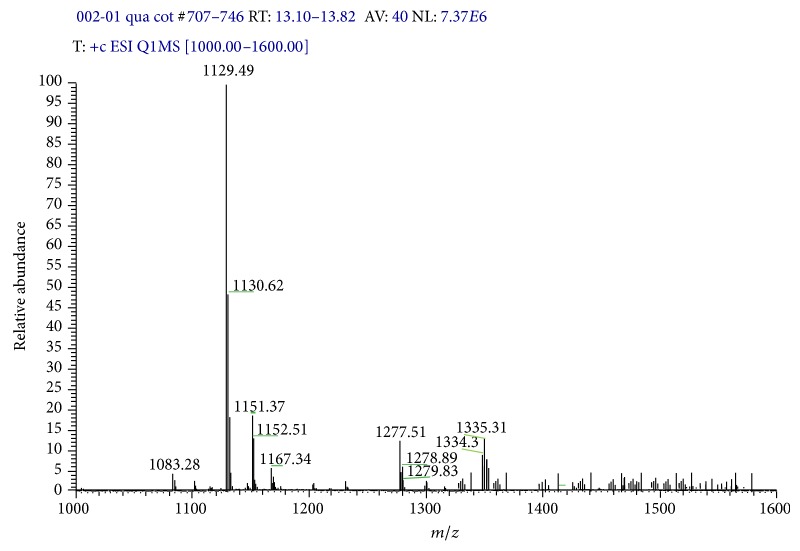
ESI-MS spectra of complex TEAC-Pb(II).

The ESI-MS of TEAC-Cr(III) and TEAC-Pb(II) were recorded in solution. Interestingly, as seen in MS spectra the ion fragments TEAC-Cr (*m*/*z* = 1177) and TEAC-Pb (*m*/*z* = 1335) appeared. Thus, these results confirmed that the ratio of TEAC with metal ions in complexes was 1 : 1. Besides, some ion fragment with higher mass also appeared, and this phenomenon can be explained that TEAC has holes space leading to MeOH molecules which have fallen into these spaces and interacted with TEAC* via* host-guest interaction (Figures 8 and 9).

### 3.3. Determination of Cr(III) and Pb(II) Based on Complexes by HAPSM

#### 3.3.1. Chosen Wavelength

**Figure 10:**
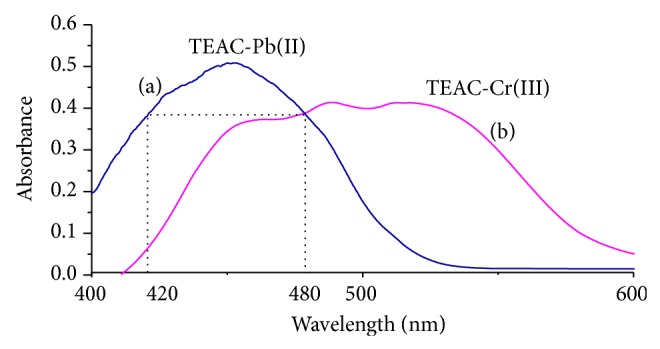
Selected wavelengths for HPSAM method at pH 10.5 (a), TEAC-Pb(II), and (b) TEAC-Pb(II).

From the absorption spectra described in Figure 10, at 420 nm and 480 nm wavelength pairs, the absorbance of the complex TEAC-Pb(II) presented similar values while the difference in absorbance of the complex TEAC-Cr(III) was observed. Therefore, the pairs of wavelengths at 420 nm and 480 nm were selected for analysis of Pb(II) ion by HAPSM with Cr(III) ion as standard addition.

#### 3.3.2. Determination of Concentration Range: Beer's Law

To determine if the concentration range is consistent with Beer's law, the graphs of absorbance versus concentration with a linear regression curve were displayed. The absorbance of the TEAC-Pb(II) and TEAC-Cr(III) complexes with concentrations from 2 × 10^−6^ to 50 × 10^−6^ M was measured at 420 and 480 nm. The result exhibited that absorbance of TEAC-Pb(II) had a linear relationship with the concentration of Pb^2+^ ion in the range of 2 × 10^−6^ to 35 × 10^−6^ M. The relationship is represented by the following regression equation: *A* = 0.0227 × *C* − 0.0166. Meanwhile, for TEAC-Cr(III), the result showed that absorbance intensity was linear with the concentration of Cr^3+^ ion from 2 × 10^−6^ to 40 × 10^−6^ M. The relationship is represented by the following regression equation: *A*_420 nm_ = 0.0103 × *C* + 0.0303 and *A*_480_ = 0.0171 × *C* + 0.0405.

#### 3.3.3. Effect of Interfering Ions

The effect of cations and anions such as Ni^2+^, Fe^3+^, Mn^2+^, Al^3+^, Cd^2+^, Co^2+^, Ni^2+^, Ca^2+^, Mg^2+^, PO_4_^3−^, Cl^1−^, and SO_4_^2−^ on the absorbance of TEAC-Pb(II) and TEAC-Cr(III) was conducted. The tolerance limit for each foreign ion is obtained when its presence at tested sample produced a variation in the absorbance of the sample greater than 5%. The results indicated that most of the cations and anions did not show any significant absorbance interference at ratio greater than 100 times. Among the cations, the Mn(II), Zn(II), and Th(IV) presented strongly interference to analyte ions. The interferences for Mn(II), Zn(II), and Th(IV) were 40, 50, and 20 times, respectively.

#### 3.3.4. Determination of Pb^2+^ and Cr^3+^ in Synthetic Samples

**Figure 11:**
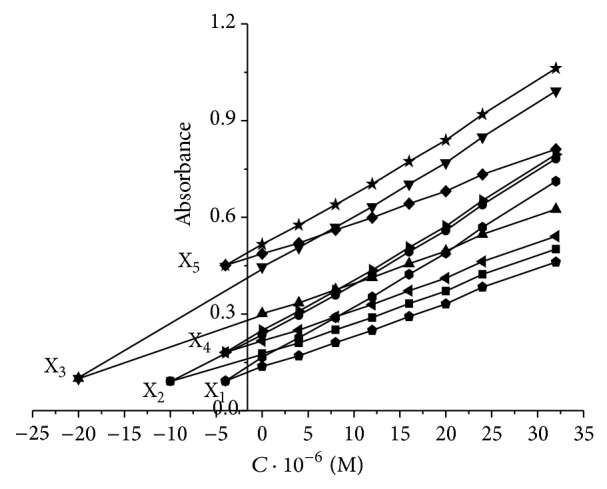
Plot of HPSAM for simultaneous determination of Cr(III) and Pb(II) ion in various mixtures: X_1_, X_2_, and X_3_ (constant concentration of Pb(II) ion and various concentration of Cr(III)) and X_4_ and X_5_ (constant concentration of Cr(III) ion and various concentration of Pb(II)).

In this system, Pb(II) and Cr(III) are the analyte and addition ion, respectively. The synthetic samples were prepared with different concentration of Pb(II) and Cr(III) named as X_1_ to X_5_ (Table 1). The calibration curves at selected wavelengths are plotted by using data of absorbencies and standard concentrations of Cr(III) ion that added to each mixture (Figure 11). As seen from Figure 11, the concentrations of Pb(II) and Cr(III) ions were calculated from a calibration graph by using *C*_H_ and *A*_H_, respectively (Table 1). Several synthetic samples were prepared to investigate the reproducibility and accuracy of the method; therefore, six replicate measurements of Pb(II) and Cr(III) were conducted under optimum condition. The results were shown in Table 1. Additionally, Figure 11 shows the H-Point Standard Addition plots for several synthetic test solutions with constant concentration of Cr(III) and Pb(II) ions, respectively. From Figure 11, the concentration of Pb(II) was found to be independent of Cr(III) ion and the concentration of Cr(III) ion was independent of Pb(II) ion.

#### 3.3.5. Application of Real Wastewater

In order to test the reality of proposed method, the plating wastewater samples were collected at the base plating in District 11, Ho Chi Minh City, Vietnam, and determined the concentration of Cr(III) and Pb(II). Samples were collected and stored in 2L plastic PE bottles and then acidified with HNO_3_ (ratio 1 : 1, v/v) to pH = 2. The water was removed and then the residue was dissolved with a mixture of HNO_3_ and HClO_4_. The resulting solution was placed in a hotplate until the white solid was obtained. The solid was diluted with distilled water and used as real sample for determination of Cr(III) and Pb(II) using HPSAM. The results were described in Table 2. The results exhibited a good agreement with those obtained from ICP-MS method. The results indicated that this highly reliable method can be utilized to analyze chromium and lead to real samples with low costs and short time.

## 4. Conclusion

The new complexes of TEAC with Cr^3+^ and Pb^2+^ were formed in basic solution. The complexation constants were 4.03 × 10^4^ and 1.20 × 10^4^ for TEAC-Cr(III) complex and TEAC-Pb(II) complex, respectively. The molar composition of the complex was confirmed as 1 : 1 by ESI-MS. Additionally, HPSAM was used for simultaneous spectrophotometric determination of chromium and leads to the spike and real sample with low cost, short time, convenience, and high accuracy. The accuracy of the method was checked by ICP-MS method. The results of this study clearly show the potential and versatility of this method for determination of toxic metal from wastewater without using organic solvents.

## Figures and Tables

**Table 1:** Results obtained by the HPSMA for the analysis of Cr(III) and Pb(II) mixtures in different concentrations.

Samples	*A*-*C* equation	*r* ^2^	Present (10^−6^ M)		Found (10^−6^ M)	
			Cr(III)	Pb(II)	Cr(III)	Pb(II)
X_1_	*A*_420 nm_ = 0.0103*C* + 0.1303 *A*_480 nm_ = 0.0171*C* + 0.1554	0.9993 0.9991	4.00	4.00	3.70 ± 0.22	4.13 ± 0.32

X_2_	*A*_420 nm_ = 0.0103*C* + 0.1703 *A*_480 nm_ = 0.0171*C* + 0.2235	0.9992 0.9989	8.00	4.00	7.75 ± 0.56	4.10 ± 0.35

X_3_	*A*_420 nm_ = 0.0102*C* + 0.2943 *A*_480 nm_ = 0.0170*C* + 0.4354	0.9995 0.9982	20.00	4.00	20.57 ± 1.25	3.71 ± 0.29

X_4_	*A*_420 nm_ = 0.0103*C* + 0.2103 *A*_480 nm_ = 0.0171*C* + 0.2389	0.9995 0.9995	4.00	8.00	4.20 ± 0.31	7.51 ± 0.62

X_5_	*A*_420 nm_ = 0.0104*C* + 0.4812 *A*_480 nm_ = 0.0169*C* + 0.5091	0.9995 0.9978	4.00	20.00	4.29 ± 0.29	19.55 ± 1.75

	LOD LOQ				1.3 4.0	1.1 3.4

**Table 2:** Results of simultaneous determination of the analysis of Cr and Pb ions in the plating wastewater (mg/L).

Samples	Content of Cr(III) mg/L		Content of Pb(II) mg/L	
	HPSAM	ICP-MS	HPSAM	ICP-MS
A1	1.45 ± 0.12	1.36	1.81 ± 0.19	1.74
A2	0.17 ± 0.02	0.16	0.62 ± 0.06	0.57
A3	0.22 ± 0.02	0.24	0.56 ± 0.06	0.65
